# Autophagy and Glycative Stress: A Bittersweet Relationship in Neurodegeneration

**DOI:** 10.3389/fcell.2021.790479

**Published:** 2021-12-23

**Authors:** Olga Gómez, Giuliana Perini-Villanueva, Andrea Yuste, José Antonio Rodríguez-Navarro, Enric Poch, Eloy Bejarano

**Affiliations:** ^1^ School of Health Sciences and Veterinary School, Universidad CEU Cardenal Herrera, CEU Universities, Valencia, Spain; ^2^ Laboratory for Nutrition and Vision Research, USDA Human Nutrition Research Center on Aging, Tufts University, Boston, MA, United States; ^3^ Servicio de Neurobiología, Departamento de Investigación, Hospital Ramón y Cajal, IRYCIS, Madrid, Spain

**Keywords:** autophagy, glycation, AGEs, neurodegeneration, aging

## Abstract

Autophagy is a fine-tuned proteolytic pathway that moves dysfunctional/aged cellular components into the lysosomal compartment for degradation. Over the last 3 decades, global research has provided evidence for the protective role of autophagy in different brain cell components. Autophagic capacities decline with age, which contributes to the accumulation of obsolete/damaged organelles and proteins and, ultimately, leads to cellular aging in brain tissues. It is thus well-accepted that autophagy plays an essential role in brain homeostasis, and malfunction of this catabolic system is associated with major neurodegenerative disorders. Autophagy function can be modulated by different types of stress, including glycative stress. Glycative stress is defined as a cellular status with abnormal and accelerated accumulation of advanced glycation end products (AGEs). It occurs in hyperglycemic states, both through the consumption of high-sugar diets or under metabolic conditions such as diabetes. In recent years, glycative stress has gained attention for its adverse impact on brain pathology. This is because glycative stress stimulates insoluble, proteinaceous aggregation that is linked to the malfunction of different neuropathological proteins. Despite the emergence of new literature suggesting that autophagy plays a major role in fighting glycation-derived damage by removing cytosolic AGEs, excessive glycative stress might also negatively impact autophagic function. In this mini-review, we provide insight on the status of present knowledge regarding the role of autophagy in brain physiology and pathophysiology, with an emphasis on the cytoprotective role of autophagic function to ameliorate the adverse effects of glycation-derived damage in neurons, glia, and neuron-glia interactions.

## Introduction: Autophagy in Brain Aging

Autophagy is a cellular cleaning process that involves the degradation of internal components through lysosomal machinery. It is a strictly regulated catabolic process that plays an important role in cell growth, development, and homeostasis by maintaining a balance between the synthesis, degradation, and subsequent recycling of cell products (Glick et al., 2010; Galluzzi et al., 2017). There are different types of autophagy: macroautophagy, microautophagy, and chaperone-mediated autophagy (CMA). All of these forms of autophagy promote the degradation of cytoplasmic components in the lysosome, but differ in the mechanisms used for selection and incorporation of the cargo into the lysosome and in the molecular determinants and steps involved in each catabolic pathway (Kaushik et al., 2010; Arias and Cuervo, 2011; Li et al., 2012). To date, mounting evidence supports the essential role of CMA and macroautophagy in maintaining proteostasis in brain tissues, while information about microautophagy remains scarce. Of note, different *in vitro* and *in vivo* models deficient for CMA support that a lack of this proteolytic capacity impacts the neuronal proteome, alters neuronal function, and enhances neuronal proteotoxicity ((Orenstein et al., 2013; Bourdenx et al., 2021b; Caballero et al., 2021) and reviewed in (Bourdenx et al., 2021a)).

**FIGURE 1:**
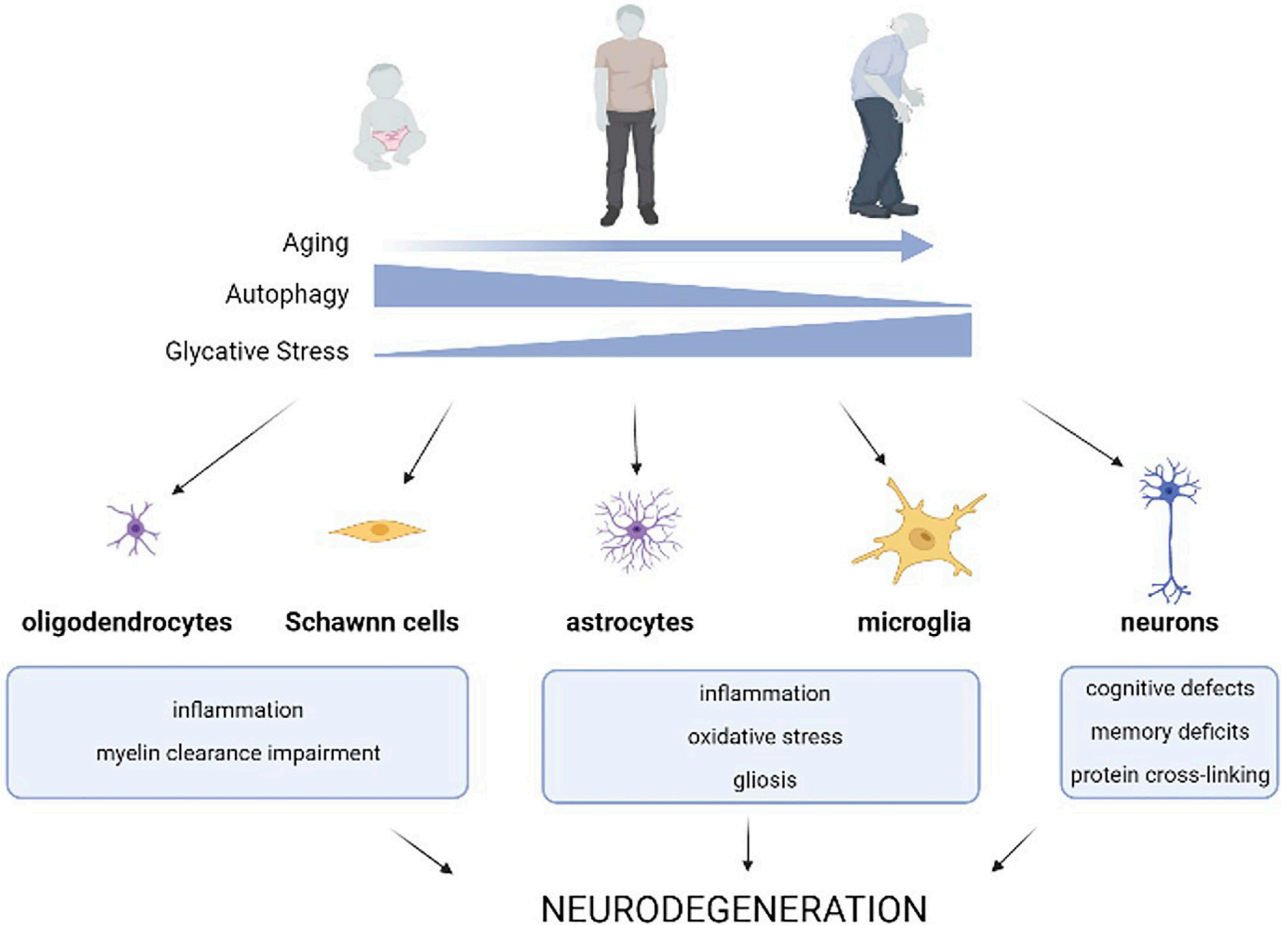
Effects of age-related autophagy dysfunction and glycative stress on brain homeostasis. Age-related decline of autophagic capacity occurs simultaneously with enhanced glycative stress in our tissues. Glycative stress is caused by reducing sugars or metabolites derived from sugar breakdown that react with different biomolecules in a non-enzymatic process called glycation. This results in an age-dependent accumulation of advanced glycation end-products (AGEs) in our tissues. Age-related autophagy dysfunction, along with glycative stress, impact the function of different cell types of the nervous system and contribute to the onset and worsening of neurodegenerative diseases.

Age-related malfunctioning of autophagy has been reported for decades, although the mechanisms behind this evidence are still poorly understood (Klionsky et al., 2021) (Figure 1). CMA age-related decline is caused by the reduced stability of the lysosomal receptor for this pathway, an instability that is further perpetuated by certain diets (Cuervo and Dice, 2000; Rodriguez-Navarro and Cuervo, 2012; Cuervo and Wong, 2014; Loos et al., 2017). In the case of macroautophagy (hereafter, autophagy), which requires the fusion of double membrane compartments (autophagosomes) with lysosomes, there are multiple molecular steps affected with age. For instance, age-dependent failure has been associated with transcriptional changes of ATG proteins that are required for trapping of the cargo, biogenesis of the autophagosomes, and maturation of lysosomal proteases (Cuervo and Macian, 2014). Defects in intracellular trafficking shown in old organisms also reduce the efficiency of autophagosome-lysosome fusion (Bejarano et al., 2018).

Autophagy plays a vital role in the cellular homeostasis of all cell types and the malfunction of this catabolic pathway is especially evident in long-lived, post-mitotic cells. This includes neural cells, which depend on an efficient proteostasis system to maintain cellular health. The role of autophagy in the CNS has been extensively described. Altered autophagic function promotes neurological protein accumulation and neuronal dysfunction and autophagy deficiency impacts axonal growth, synaptic plasticity and dendritic formation and pruning (Figure 1). For this reason, autophagy has become a promising pharmacological target for reducing the levels of toxic substances that accumulate in the brain of patients. During the last several years, a set of drugs that activate autophagy such as rapamycin, resveratrol, trehalose, and spermidine have been assayed to prevent or ameliorate the progression of certain neurological diseases (Panda et al., 2019).

## Glycative Stress and Autophagy: A Pathological Axis in Neurodegeneration?

**FIGURE 2:**
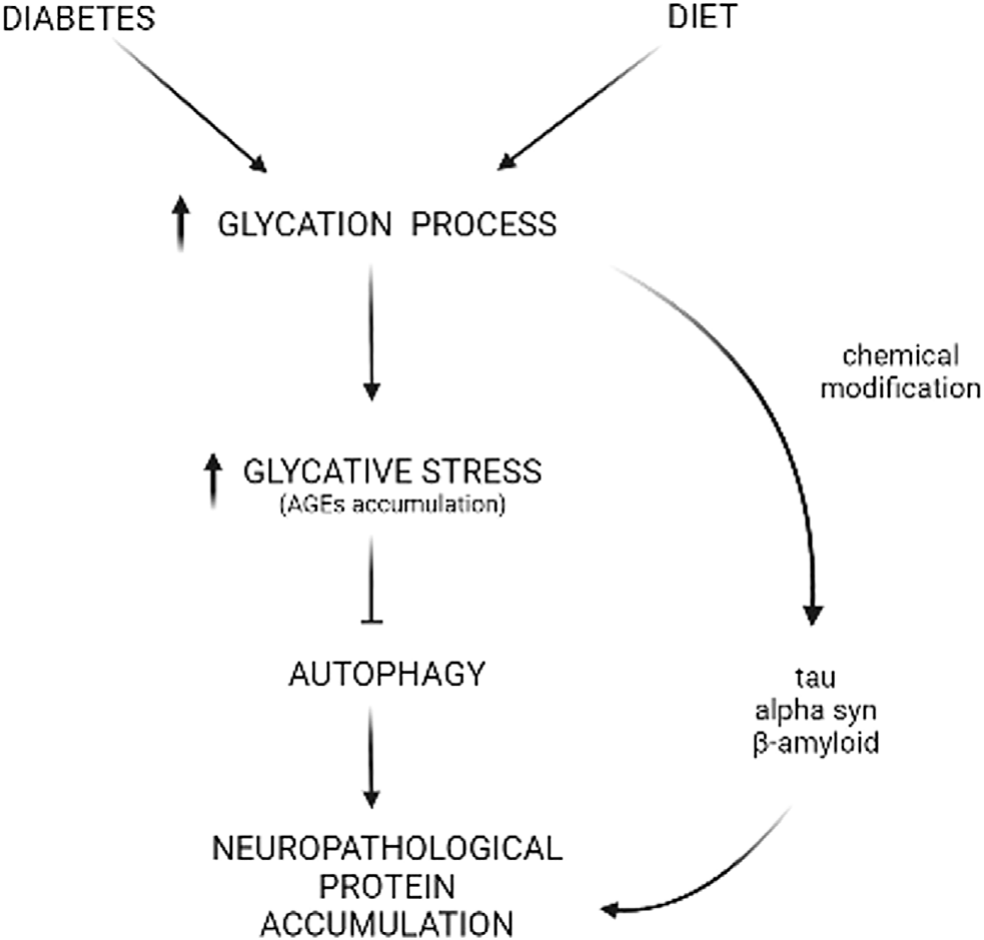
Glycative stress and deficient autophagy behind neuropathological protein accumulation. Diabetes or high-carbohydrate diets increase the glycation process, leading to AGEs accumulation that includes CML, pentosidine, MG-H1, and more. Reactive sugars or metabolites can react with autophagic proteins, leading to a deficient autophagic function that, ultimately, impacts the turnover of proteins involved in neurological disorders such as alpha-synuclein (α-syn), tau, or beta-amyloid (Aβ). Also, this glycative chemical modification can occur directly in the sequence of these proteins, resulting in insolubilization and aggregation.

Glycative stress refers to a type of stress caused by reducing sugars (glucose, fructose, and galactose) or metabolites derived from sugar breakdown that react with different biomolecules (proteins, lipids, and nucleic acids) in a non-enzymatic process called glycation (Rabbani and Thornalley, 2015). Advanced glycation end-products (AGEs) are the result of this process, and these glycated biomolecules undergo changes in properties such as location, solubility, and function (Rowan et al., 2018). In proteins, glycation promotes cross-linking, loss of function, and aggregation. Altogether, the changes made to proteins negatively impact cellular metabolism. Given that glycation is dependent on sugar concentration, these harmful glycation adducts are produced at a higher rate under conditions such as hyperglycemia, consumption of high glycemic diets, and diabetes. These conditions exacerbate the detrimental impact of AGEs in tissue and organ fitness (Uribarri et al., 2015) (Figure 2).

Although age-associated AGEs accumulation has been reported in multiple tissues (Uchiki et al., 2012), the deposition is tissue and age-dependent (Figure 1). The tissues that are most susceptible to glycative stress-induced toxicity are those with low regenerative capacity and highly differentiated cellular components. This signifies that ocular and brain tissues are highly vulnerable to the effects of glycative stress (Uchiki et al., 2012; Aragonès et al., 2020a). Due to this vulnerability, AGEs play a crucial role in the pathogenesis of different age-related disorders associated to these tissues such as cataracts, age-related macular degeneration, and neurodegenerative diseases (Semba et al., 2010; Bejarano and Taylor, 2019). Compelling literature also indicates that glycative stress contributes to the pathogenesis of several CNS disorders and diseases including peripheral diabetic polyneuropathies, Alzheimer’s, Parkinson’s, Huntington’s, Creutzfeldt-Jakob disease, and amyotrophic lateral sclerosis (Münch et al., 2012). High levels of AGEs are found in Lewy bodies of subcortical neurons in Parkinson’s patients, as well as in the plaques of patients suffering from Alzheimer’s disease (Münch et al., 2000; Guerrero et al., 2013). Dietary-induced glycative stress through the consumption of a high glycemic index diet or high-fructose diet has also been found to lead to AGEs accumulation in the mouse brain (Uchiki et al., 2012; Mastrocola et al., 2016). Exposure to exogenous AGEs (formed during heating and irradiation of foods) or high sugar diets that accelerate the intracellular synthesis of AGEs were shown to impact brain function. A diet high in exogenous AGEs accelerated Aβ deposition in an Alzheimer mouse model and was associated with impaired learning and memory along with mitochondrial dysfunction (Cai et al., 2014; Lubitz et al., 2016; Akhter et al., 2020). Interestingly, anti-glycative activity found in dietary phytochemicals of berry fruits might have benefits that preserve cognitive function (Thangthaeng et al., 2016). In fact, high levels of dietary AGEs were associated with faster rate of memory decline in non-demented young elderly (West et al., 2014) and clinical trials are currently testing if dietary reduction of AGEs could lead to improved cognition in elderly with type 2 diabetes. Previous dietary interventions have also shown improvements in participants with mild cognitive impairment (Lotan et al., 2021). Overall, *in vitro* and *in vivo* experiments in different animal models support the hypothesis that strategies for lowering AGEs deposition are neuroprotective (Bélanger et al., 2011; Chaudhuri et al., 2018).

Information regarding how glycative stress contributes to the onset and worsening of neurodegenerative diseases is scarce. However, recent literature suggests that glycation interferes with vital brain function processes. For example, there is evidence that glycative stress could trigger neuronal differentiation defects in neural stem cells and impact neurite regeneration (Bao et al., 2020). Some reports also suggest that there is a modification of dopamine due to glycation precursors (Deng et al., 2012). Intracellularly, glycation promotes the cross-linking of neuropathological proteins such as amyloid β, tau, and α-synuclein. This cross-linking gives rise to neuropathological protein insolubility and decreases the removal of toxic oligomers (Guerrero et al., 2013; Li et al., 2013; Liu et al., 2016; Vicente Miranda et al., 2017). Extracellular AGEs induce reactive gliosis and the activation of the NF-κB proinflammatory pathway, all of which lead to eventual neuronal degeneration (Wang et al., 2013). Of note, the existence of cell-dependent mechanisms that help combat AGEs accumulation in the brain have been reported. For example, higher glyoxalase system activity has been seen in primary mouse astrocytes, as compared to neurons (Bélanger et al., 2011). The glyoxalase system catalyzes the detoxification of glycation precursors (Aragonès et al., 2020b; Aragonès et al., 2021) and a weaker defense mechanism against glycation-derived damage in neurons leads to a higher susceptibility for glycation-induced neurotoxicity.

In order to avoid the glycation-derived perniciousness, our cells maintain non-toxic homeostatic levels of AGEs by deploying a battery of anti-glycation mechanisms that include detoxifying pathways that limit the synthesis of AGEs (glyoxalase system, Parkinson-associated protein DJ-1, aldehyde dehydrogenases, aldo-keto reductases, and acetoacetate degradation) and proteolytic AGEs elimination through UPS and autophagy (Aragonès et al., 2021). Due to the irreversible nature of the glycation process, AGEs removal is the last line of defense against associated glycation-derived tissue malfunction (Rowan et al., 2018; Taylor and Bejarano, 2022). Cytotoxic AGEs are degraded either in the proteasome through the ubiquitin-proteasome system (UPS) or in the lysosomal compartment through autophagy. The redundancy of these clearance routes is not well-understood, although it is thought that UPS mainly removes soluble glycated proteins while autophagy destroys insoluble glycated proteins and aggregates (Taylor, 2012). Pharmacological and genetic inhibition of autophagy increases vulnerability to glycative stress, while the enhancement of autophagy lowers the levels of AGEs (Uchiki et al., 2012; Takahashi et al., 2017; Aragonès et al., 2020a). It is also unclear how molecular determinants trigger the degradation of AGEs, although ubiquitination of glycated proteins and recognition by corresponding autophagic receptors appears to participate in the degradation of AGEs (Uchiki et al., 2012; Aragonès et al., 2020a). Interestingly, the UPS inhibitor lactacystin, injected in the hippocampus of young rats did not cause an accumulation of AGEs. However, high levels of AGEs deposition were detected in aged rats after the same UPS inhibition (Aragonès et al., 2020a). This indicates that age is a major variable for the nature of AGEs deposition. Emerging evidence also suggests that the intensity and duration of glycative stress are other factors that should be taken into consideration because of their potential impact on the proteolytic systems that degrade AGEs. Chronic or acutely high levels of such stress could lead to direct glycation of components in the UPS or autophagy, inactivating the systems and triggering proteotoxicity and death of cellular brain components (Queisser et al., 2010; Aragonès et al., 2020a). It creates a vicious cycle: high glycative stress inhibits autophagic capacity, leading to insufficient clearance of AGEs which, in turn, enhances glycative stress. However, the threshold of sensitivity for the proteolytic capacity of different brain components to become compromised by glycation remains unknown (Figure 2).

## Glycative Stress and Neuroprotective Role of Autophagy in Neurons-Glia Interactions

Interactions between glial and neuronal cells play a crucial role in brain physiology and in the degeneration of the aging brain. Glial cells represent approximately 90% of the cells in the brain and are key regulators of neuronal homeostasis and function. The interaction with neurons may be direct (e.g. through nanotubes or connexin-based intercellular channels) or indirect (e.g. secretion of molecules or vesicles). More importantly, all these types of interactions are modulated by autophagy.

The exchange of metabolites between neurons and glial cells is immense, and both autophagy and glycative stress influence the exchange. As stated above, autophagy responds to nutrient scarcity, and in the brain, autophagy is crucial in glial cells because it provides nutrients and other metabolites to neurons. This allows the neurons to specialize and develop the capability to conduct nerve impulses (Kulkarni et al., 2020). Traditionally, the role of providing nutrients and antioxidants to neurons, important in conditions like strokes or epilepsy, has been attributed to astrocytes (Bejarano and Rodríguez-Navarro, 2015; Liu et al., 2018; Perez-Alvarez et al., 2018), but the role of other cell types, like oligodendrocytes and tanycites, is still being deciphered (Belgrad et al., 2020; Lhomme et al., 2021). AGEs are by-products of this metabolite generation, so glial cells tend to have higher detoxification capabilities than neurons, both enzymatically and through the autophagy-lysosomal system (Bélanger et al., 2011; Liu et al., 2018; Perez-Alvarez et al., 2018). As a consequence of the AGEs by-products, glial cells, specifically astrocytes, have been found to express high levels of the DJ-1/Park7 gene which encodes for a deglycase enzyme that counteracts glycation (Bandopadhyay et al., 2004). Mutations in the protein DJ-1 cause autosomal recessive forms of Parkinson’s disease (PD). Although the deficiency of DJ-1 has been associated with higher values in several glycative stress parameters of *in vivo* and *in vitro* models (concentrations of methylglyoxal and AGEs, including carboxymethyllysine) (Pantner et al., 2021), the capacity of DJ-1 to combat glycative stress *in vivo* is under debate and its role is not fully understood ((Mencke et al., 2021). Reduced DJ-1 activity might contribute to accumulation of glycated α-synuclein and its aggregates (Sharma et al., 2019). Furthermore, consistent with the pathogenic role of glycative stress in PD, higher levels of AGEs were found in plasma and in the periphery of Lewy bodies of PD patients (Sharma et al., 2020). In alignment with this evidence, glycation was reported in different Parkinson’s models, and diets that enhance glycative stress are shown to increase susceptibility to PD features such as motor symptoms and α-synuclein aggregation (reviewed in (Videira and Castro-Caldas, 2018). Whether deficient DJ-1 plays a role in the onset of PD remains an enigma.

The generation of tunneling nanotubes connecting multiple neurons or connecting neurons and glial cells, as well as transferring toxic aggregates or mitochondria between them, are mediated by autophagy (Chastagner et al., 2020; Walters and Cox, 2021). The guidance of the nanotubes between neurons and astrocytes is mediated by the receptor for advanced glycation end products, otherwise known as RAGE (Sun et al., 2012).

Neuroglial interactions are regulated through a flux of information that is communicated *via* connexin-based intercellular channels. These channels are permeable to multiple biomolecules including second messengers, glucose, amino acids, nucleotides, and ions. Connexins, the structural blocks of these channels, are endogenous inhibitors of autophagosomal biogenesis in basal conditions (Bejarano et al., 2014). Conversely, upon experiencing different types of stressors, this inhibition is released and autophagy contributes to the turnover of connexins in astrocytes and neurons (Bejarano et al., 2012; Sun L. et al., 2015; Sun L. Q. et al., 2015; Chen et al., 2017). The inhibition of connexin-based intercellular communication prevents glycation-derived toxicity in astrocyte cultures. Astrocyte viability depends on gap junction function and, although the exact molecular mechanism is not well-understood, glutamate uptake activity is a target of glycation, and glycative stress-derived impairment involves glutamate excitotoxicity (Hansen et al., 2017). *In vitro* glycative stress was also shown to increase RAGE expression and levels of connexins in cardiomyoctes and activated human microglial CHME-5 cells (Shaikh et al., 2012; Yu et al., 2013). However, the literature is controversial and other studies have reported downregulation of connexins in human aortic endothelial cells and in human hepatome cells while in the presence of glycative stress (Lin et al., 2010; Wang et al., 2011).

Indirect cell-to-cell communication among brain cells occurs through the secretion of extracellular microvesicles (EVs). Along with multiple types of synaptic vesicles, there is a great variability of vesicles in the nervous system including exosomes, microvesicles and apoptotic bodies. The methods of vesicle extraction and isolation are very limited and the isolation of different subtypes of EVs in the nervous system is challenging, further complicating the analysis of brain EVs. The molecular composition (proteins, lipids, metabolites, RNAs, etc.) of EVs vary with cell type, nutritional and oxidative status, and age (Carpintero-Fernández et al., 2017). Due to this variability, the vesicles have distinct roles in brain diseases, whether they are deleterious or protective (Kučuk et al., 2021; Zhang et al., 2021). For example, small EVs from young cells are able to rejuvenate different tissues in old mice and their role as a tool for the delivery of specific therapies is being thoroughly investigated (Kumar et al., 2020; Rodriguez-Navarro et al., 2020). Regarding the EV’s relationship to glycation, RAGE is enriched in plasma EVs derived from neurons, and different AGEs precursors (such as glyoxal and methylglyoxal) have been proposed as biomarkers of neurodegeneration due to their presence in neuronal derived EVs (Haddad et al., 2019). On the contrary, modified anti-RAGE EVs have been proposed as a treatment for brain stroke neurodegeneration (Kim et al., 2021).

The release mechanism of these vesicles also varies and secretory autophagy, an unconventional release mechanism, is involved in the extrusion of α-synuclein, tau, or amyloid-beta aggregates associated with neurodegenerative diseases (Majerova et al., 2014; Wang et al., 2017; Gonzalez et al., 2020). The extracellular presence of these aggregates allows for the propagation of the disease (Caballero et al., 2021). These aggregates are usually enriched in AGEs (Uchiki et al., 2012). Recent evidence demonstrates that neurons extrude not only protein aggregates, but also oxidized organelles in membrane-bound vesicles called exophers that are subsequently phagocytosed by other glial cell types (Melentijevic et al., 2017). Microglia is the traditional cell type within the brain, recognized for its role of cleaning unwanted materials. However, astrocytes also have significant phagocytic activity (Majerova et al., 2014; Asai et al., 2015; di Domenico et al., 2019), and the autophagic lysosomal system in every cell type is involved in the degradation of these preformed aggregates. Interestingly, glycative stress disrupts the blood brain barrier and promotes the secretion of tight junction proteins onto EVs (Rom et al., 2020). Neuronal cells treated with glycating reagents also experience changes in neuronal derived-extracellular vesicles (Haddad et al., 2019).

All this evidence suggests that both autophagy and glycative stress play a major role in neuron-glia interactions. Although the stimulation of autophagy and the clearance of AGEs are being actively studied as therapies for neurodegenerative diseases (Brás et al., 2019; Frandsen et al., 2020; Kocak et al., 2021), information about the relationship between autophagy and glycative stress on specific brain components is still limited and it should be taken into account across all different cells of the nervous system.

## Concluding Remarks

Autophagy is a key quality control mechanism for brain and autophagic malfunction that is linked to many neurodegenerative diseases. When glycative stress levels are moderate, autophagy is active and removes toxic, glycated biomolecules to counteract glycative burden. However, higher levels of glycative stress seem to directly impact autophagic function. This is an issue for aging individuals because glycative stress creates an extra compromise on the already present, age-related decline of autophagy. Different cellular potentials for dealing with glycative stress in the brain have to be considered for future interventions. A better understanding of the process and the identification of glycated ATGs is imperative for designing strategies to ameliorate the burden of glycative stress. A significant effort should also be made to identify nutritional compounds and/or dietary interventions that maintain homeostatic levels of AGEs. Lowering glycative stress could assist with the preservation of autophagic capacity despite age and decrease the aggregation of neuropathological proteins, extending brain function and health span.
